# Social competencies of children with disinhibited social engagement disorder: A systematic review

**DOI:** 10.1002/jcv2.12226

**Published:** 2024-03-08

**Authors:** Claire Davidson, Shahela Islam, Enrico Venturini, Anja Lowit, Christopher Gillberg, Helen Minnis

**Affiliations:** ^1^ University of Glasgow Academic CAMHS West Glasgow Ambulatory Care Hospital Glasgow UK; ^2^ University of Strathclyde, Speech and Language Therapy Glasgow UK; ^3^ Gillberg Neuropsychiatry Service University of Gothenburg Gothenburg Sweden

**Keywords:** child maltreatment, disinhibited social engagement disorder, social competency, social relationships, social skills

## Abstract

**Background:**

Children with Disinhibited Social Engagement Disorder (DSED) have specific difficulties with indiscriminate sociability, yet little is known about their broader social competencies as DSED tends not to be identified within samples in the wider ‘maltreatment literature.’

**Aim:**

To systematically review the literature to determine the social competencies of children with DSED.

**Methods:**

A comprehensive search following PRISMA guidelines was undertaken using PsycINFO, Medline, Embase, and Cumulative Index to Nursing & Allied Health.

**Results:**

From a total of 553 articles, 16 studies were selected and critically evaluated. Children with DSED were consistently reported to have poorer social competencies than non‐maltreated peers and environmental controls. Greater peer problems were consistently found, and they may present with poor self‐esteem/concept related to social acceptance. Findings regarding social interaction/communication skills were mixed.

**Limitations:**

50% of studies were of moderate quality due to sampling and possible confounding variables.

**Conclusion:**

Children with DSED present with social relationship problems, beyond the core symptoms of the disorder, but the relative impact of co‐occurring neurodevelopmental conditions is not yet clear. In addition, pragmatic language and communication skills require further research.

**Key points**

- What's known: Maltreated children may be more susceptible to social relationship problems, but Disinhibited Social Engagement Disorder (DSED) is often not identified within these samples, despite maltreatment being considered part of the disorder's aetiology.
- What's new: Children with DSED demonstrate poorer social competencies and greater relational conflicts than typically developing peers, and in some cases, environmental controls. Possible pragmatic language deficits also require further investigation.
- What's relevant: All professionals working with maltreated children should consider assessment for DSED *and* consider impact of impaired social functioning in daily participation, especially as DSED is persistent over time. Co‐occurring neurodevelopmental conditions are also frequently reported and requires further research regarding social functioning and later outcomes.

## INTRODUCTION

Childhood maltreatment (abuse or neglect) is associated with social relationship difficulties and/or poorer quality relationships across the lifespan (Doyle & Cicchetti, 2017; Flynn et al., 2014; Goemans et al., 2023; Shonk & Cicchetti, 2001). Yet the mechanisms underpinning such problems are complex.

Attachment relationships play a crucial role in the early development of social relationships through the dyadic process of infant signalling and parental sensitivity to child behaviours (Ainsworth et al., 1974; Bowlby, 1982, 1988). This ‘serve and return’ like interaction helps build neural pathways in the developing brain (Ilyka et al., 2021), and supports development of early skills such as joint attention, which are important for later language and reciprocal social interaction (Bottema‐Beutel, 2016; Carpenter et al., 1998; Markus et al., 2000).

As a multi‐faceted concept social functioning is difficult to measure but in childhood, social competency tends to relate to peer acceptance/rejection, pro‐social skills/skill deficits, self‐regulation and ability to navigate social conflict (John, 2001).

Children who have experienced early maltreatment, however, are more susceptible to developing insecure and disorganised attachments (Bowlby, 1973, 1982; Pickreign Stronach, et al., 2011), or attachment disorders (Minnis, 2013; Zeanah et al., 2016). Social skill deficits such as poor play and joint attention, language delay, poorer identification of non‐verbal cues and deficits in facial recognition of emotions have been reported (Carr et al., 2020; Culp et al., 1991; Law & Conway, 1992; Sheaffer, Golden, & Averett, 2009; Sheaffer, Golden, & Averett, 2009). Greater peer conflicts, bullying or victimisation are associated with maltreatment (Yoon et al., 2021; Goemans et al., 2023; Humphrey's et al., 2018; Guyon‐Harris, Humphreys, Fox, et al., 2019), as well as lower self‐esteem (Cederbaum et al., 2020; Seim et al., 2022), risk of mental health problems and risky or ‘problem’ behaviour (Carr et al., 2020; Humphrey's et al., 2018).

McCrory et al. (2022) hypothesised that maltreated children may be more susceptible to cumulative stress because of factors discussed above, and, additionally, because social networks of maltreated children may diminish due to poorer social competencies and missed opportunities to build social relationships. Gajwani and Minnis (2023) argue that an important element may be the interaction of co‐occurring neurodevelopmental conditions, as maltreated children are at higher risk of presenting with one or more neurodevelopmental conditions and/or maltreatment associated disorders (Dinkler et al., 2022; Minnis, 2013).

We are particularly interested in the social competencies of children with the maltreatment‐associated disorder, DSED, as these children have specific relational problems, inherent to the diagnosis of the disorder. The core symptoms of DSED are indiscriminate sociability and poor social boundaries, which occur in the context of maltreatment (DSM‐5, American Psychiatric Association, 2013).

The term DSED is a relatively recent change to the nomenclature, which occurred with the advent of DSM‐5. Within previous diagnostic classifications, DSED was known as the disinhibited sub‐type of Reactive Attachment Disorder (d‐RAD) (DSM‐IV, American Psychiatric Association, 2000), or as Disinhibited Attachment Disorder (DAD) in the European equivalent, ICD‐10 (World Health Organisation, 1993). At that time, the presumed aetiology and lack of preferential selection of primary caregivers suggested that DSED may be a disorder of attachment. This changed as a body of evidence demonstrated that core features of DSED persisted, despite children developing secure attachments once placed with foster/adoptive families (Lyons‐Ruth et al., 2009; Minnis et al., 2007; O’Connor et al., 2003; Zeanah et al., 2005). In DSM‐5, the name DSED was introduced to better reflect the core problems of social‐relatedness and DSED is now a *separate* disorder to Reactive Attachment Disorder, (DSM‐5) (N.B. despite change of name, the core *symptoms* of DSED have not changed and remain as described under previous nomenclature).

### Overview of Disinhibited Social Engagement Disorder

Disinhibited Social Engagement Disorder behaviours were first reported among children adopted from severely deprived international institutions (Chisholm, 1998; O’Connor et al., 2000; Rutter et al., 2007; Smyke et al., 2002; Tizard & Rees, 1975; Zeanah et al., 2002), and the Bucharest Early Intervention Project (Smyke et al., 2009, 2012) and the English and Romanian Adoptees Study (O’Connor et al., 2000; Rutter et al., 2007; Sonuga‐Barke et al., 2017) were inspirational in demonstrating the childrens' needs, the possibility of positive developmental growth, and set the scene for better understanding of DSED.

Disinhibited Social Engagement Disorder has since been reported in community samples of maltreated children (Kay et al., 2016; Kay & Green, 2013; Minnis et al., 2013; Scheper et al., 2019; Seim et al., 2021). In one population study of 1646 children in a deprived UK urban area, 12 cases were diagnosed with DSED, suggesting an estimated prevalence of just less than 1% (0.72) (Minnis et al., 2013). Scheper et al. (2019) found that in a community sample of 124 children, 38% (*n* = 47) presented with DSED and symptoms were still present 4 years later in 57% of those children. Of note, when associations with neurodevelopmental conditions and environmental factors were investigated, Attention‐deficit/hyperactivity Disorder, (ADHD) was the only variable associated with persistence of DSED. This latter finding is interesting, given the recent preliminary research which suggests that different dimensions of maltreatment that is, threat versus deprivation may have different effects on neurodevelopmental outcomes (Ellis et al., 2022; Sheridan & McLaughlin, 2014). Disinhibited Social Engagement Disorder is thought to be associated with severe neglect, (social‐emotional, in particular) (Zeanah & Gleason, 2015; DSM‐5, 2013; Oliveira et al., 2012) and there is some evidence to suggest that severe deprivation, as opposed to threat, is associated with changes in cortical and white matter in the brain, reduced cognitive ability and negative effects on executive functioning (McLaughlin et al., 2017). While, DSED has been found to overlap with other neurodevelopmental conditions, such as Autism (Dinkler et al., 2017; Mayes et al., 2017; Rutter et al., 1999), it is ADHD, or symptoms of ADHD, which appear more prevalent (Bruce et al., 2009; Pears et al., 2010; Bos et al., 2011; Seim et al., 2022). Even in early adulthood, when both Autism and ADHD were found to co‐occur with persistent DSED, it was the interplay with ADHD which was associated with poorer functional outcomes (Kennedy et al., 2017).

Nevertheless, the scarcity of research with children with DSED is still a concern (Zeanah et al., 2016). In other social impairment disorders, such as Autism, our wealth of knowledge (Carter et al., 2005) gives parents and clinicians better understanding about the difficulties that children experience. Such knowledge is crucial in assessment and case management, in reducing stress, supporting relationships and school functioning. Furthermore, there is ongoing concern regarding differential diagnosis of DSED from Autism (Davidson et al., 2015; Davidson, Minnis and Moran, 2022; Mayes et al., 2017; Moran, 2010), yet lack of knowledge about DSED and broader social problems makes it *even* more difficult for clinicians to untangle possible overlaps.

## METHODS

### Aims & research question

A scoping search of the literature revealed no synthesis of data regarding the social relationships of children with DSED. To address the gap in knowledge, we aimed to systematically review the literature to assess the social competencies (interpersonal relations, social skills, conflicts and perceptions of self) of children with DSED. We proposed the following research question:

1. Do children with DSED demonstrate impaired social competencies, beyond the core problem of indiscriminate behaviours?

### Search strategy

Following the Preferred Method of Reporting of Systematic Reviews guidelines (PRISMA) (Liberati et al., 2009) four electronic databases were searched: PsycINFO (1872‐present), Embase (Ovid, 1947‐present update daily), Cumulative Index to Nursing & Allied Health (1973‐present) and Medline (Ovid, 1946 to January Week 3 2017 & OVID 1946 to March 28, 2023). Studies were limited to English.

The search shown below exemplifies the search strategy:

Example phase 1 search of PsycINFO using subject headings and key words.

1. DSED (major concept) OR Attachment Disorder (explode).
2. Key words: Disinhibited Social Engagement OR DSED OR Attachment Disorder OR Indiscriminate friendl* OR Overfriendl* OR Over friendl* OR Indiscriminate Sociability
3. Combine 1 & 2 using “AND.”
4. Social competence (major concept) OR Interpersonal relationships (major concept) OR relationship quality (major concept) OR interpersonal interaction OR social adjustment (major concept), OR social interaction (major concept), OR social skills (major concept) or social communication (major concept),
5. Social relationship* OR social interaction OR interpersonal relations* OR social skills OR interpersonal skills OR interpersonal interactions OR social communication, OR interpersonal communication OR pragmatic language.
6. Combine 4 & 5 using “AND.”
7. Combine 3&6

Phase 2: We examined DSED synonym keyword searches individually by title and abstract, as some seminal studies have a broader focus for example, prognosis post‐institutionalisation, yet still have relevance.

Findings were appraised by title and abstracts. Selected studies were read in full. CD and SI reviewed a third of abstracts jointly to calibrate the process then both individuals reviewed half each of the remaining articles. Uncertainties were discussed at conference until agreement was reached.

### Inclusion and Exclusion Criteria

Inclusion Criteria:

1. Study sample included children up to 18 years with a diagnosis of either DSED, RAD (DSM‐IV) or dRAD (DSM‐IV), or DAD (ICD‐10) or core symptoms that is, indiscriminate friendliness with strangers in the context of maltreatment.
2. Paper discussed social competencies or impact on social relationships, social skills (verbal/nonverbal) or concepts such as self‐esteem in relation to social functioning.
3. Studies used a standardised tool, observation or qualitative methods.

Exclusion Criteria:

1. Sample included maltreated children but did not identify DSED symptoms.
2. The study was about attachment patterns that is, secure/insecure attachments.
3. Thesis abstract, case study only or review.
4. Not available in English.

Due to the change in nomenclature, it was necessary to include studies in which the population was defined using the previous DSM‐IV terminology (RAD, disinhibited RAD, indiscriminate friendliness)/ICD‐10 (Disinhibited Attachment Disorder (DAD)). However, this has no impact on the integrity of the results as the key symptoms of the disorder did not change with the DSM‐5 re‐classification. In some cases, the authors did not directly discriminate between sub‐types of RAD (DSM‐IV), but these studies were not excluded to ensure relevant data was not missed. This is methodologically justified for two reasons, 1. RAD (DSM‐5) (the inhibited form) on its own has been demonstrated to be rare in the population (Minnis et al., 2013; Zeanah, 2000); it is DSED that is persistent, 2. RAD is a separate disorder to DSED (Gleason et al., 2011; Zeanah & Gleason, 2015; Zeanah, 2016; APA DSM‐5, 2013; Lehmann et al., 2020) with separate symptomology, despite its shared aetiology. Thus, if RAD symptoms were present, these would be considered as an additional but co‐existing problem. In discussion of the results, we have used the term DSED^RAD^ to identify these older studies which likely contain mainly DSED cases but may include some cases of RAD. Otherwise, the use of terms DSED and RAD refer to the current DSM‐5 diagnostic criteria.

The Crowe Critical Appraisal Tool, V1.4 (Crowe et al., 2011) was used to rate study quality because it can be applied to both quantitative and qualitative methodologies. A score of <20 is considered low quality, 20–29 moderate quality, and 30–40 high quality. CD, SI and HM rated a third of the included full text articles independently and discussed findings jointly.

## RESULTS

**FIGURE 1:**
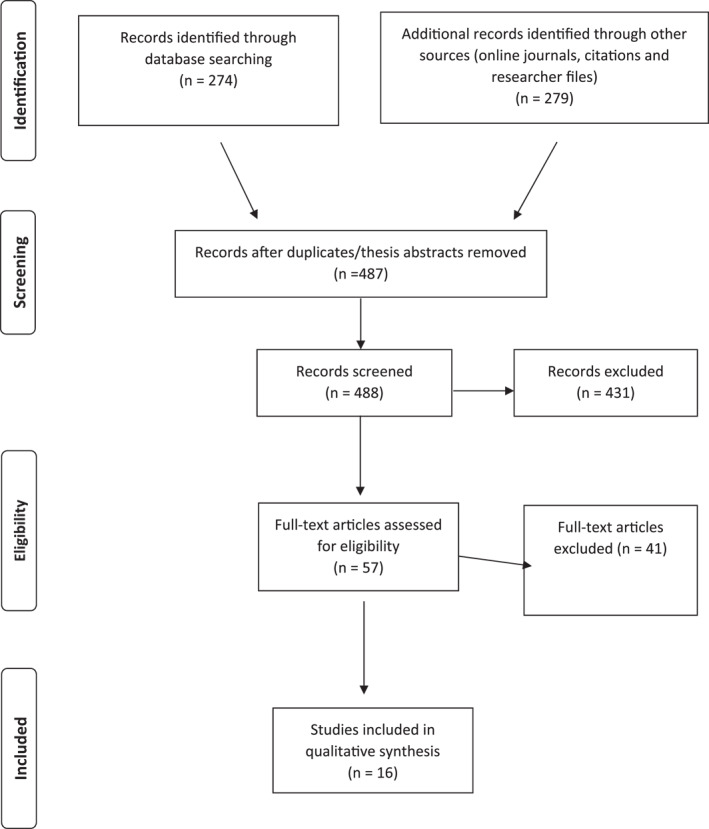
Preferred Method of Reporting of Systematic Reviews guidelines (PRISMA) flow diagram.

A total of 553 abstracts were identified, 496 removed and 57 studies read in full, of which, 41 were ineligible (see Figure 1).

Sixteen studies were included (50% of high quality and 50% of moderate quality) (see Table 1).

**TABLE 1:** Study details.

Authors/Date	Study design	Investigative focus of study	Participants (*n*, age range, recruitment)	Measures (related to social functioning only	Outcomes (pertaining to social relationships/social function only)	CCAT score
**1. Social competence**						
Millward et al. (2006)	Quantitative cross sectional	Investigate symptoms of DSED^RAD^ in children and related outcomes	82 families of children in foster care, residential care or special education schools, (age range 4–16 years).	Strengths and difficulties questionnaire (SDQ) via parent report.	Children with DSED^RAD^ symptoms were likely to score highly on all sub‐scales of the SDQ and significantly more poorly than controls.	29 Moderate
			Control group: 125 families from local nurseries and schools and 231 families recruited from local general practices, within a similar socio‐economic status, (age range 5–16 years), matched on age and gender.			
			DSED^RAD^ symptomology established via the standardised reactive attachment disorders questionnaire (caregiver report).			
Pritchett et al. (2013)	Quantitative	To describe the characteristic of children with DSED^RAD^	22 children (6–8 years old) with definite or suspected DSED^RAD^ from population screening of 1600 children.	Strengths and difficulties questionnaire (SDQ), completed by care givers and teachers.	SDQ: 75% of children with definite or suspected DSED^RAD^ scored within the abnormal range.	28 Moderate
			Symptoms of DSED^RAD^ were identified via triangulation of standardised measures of caregiver report (relationships problems questionnaire, child and adolescent psychiatric assessment ‐RAD and development and wellbeing assessment) and child observation (waiting room observation). Diagnoses were made using DSM‐IV criteria by clinicians in the research team.	Social skills improvement system which assesses social skills, problem behaviours and academic competence.	SSIS: 10/22 children scored below average, compared to american norms.	
						
Giltaij et al. (2016)	Quantitative cross sectional	Tested whether children with RAD or DSED had lower adaptive functioning (which included socialisation) than peers without RAD or DSED.	Total sample of 55 children with intellectual disabilities, mean age 10 years, mean IQ 72.1.	Vineland screener 0–12.	Vineland: DSED & mixed DSED/RAD group scored significantly lower than peers for socialisation.	28 Moderate
			1/55 had DSED symptoms; 6/55 children mixed RAD/DSED. Comparison group, *n* = 45/55.	The developmental behaviour checklist (DBC)	DBC parent: Mixed DSED/RAD & DSED only group more disruptive and anti‐social behaviour than peers. DBC teacher: More emotional disturbance in children, without DSED or mixed DSED/RAD.	
			DSED & RAD symptoms were based on the list of behavioural signs of disturbed attachment in young children following observation of parent‐child interactions.			
Guyon‐Harris, Humphreys, Fox, et al. (2019)	Quantitative, prospective cohort study	Investigated the association between symptoms of DSED in early childhood and social competency in adolescence, across multiple domains.	136 Romanian children from the bucharest early intervention project (BEIP).	The authors created a composite of competent functioning based on 7 domains (family relationships, peer relationships, academic performance, physical health, mental health, substance use and risky behaviour); information was gathered via items from the following standardised measures:	Children with *more* symptoms of DSED were significantly less likely to meet threshold for social competency at age 12 years.	34 High
			Caregiver reports examined at 4 time points (30, 42 and 54 months and 12 years).	The social skills rating system.	Children who received an *early* diagnosis of DSED (before 54 months) were significantly less likely to be classified as socially competent.	
			DSED symptoms were investigated via the standardised disturbances of attachment interview at all 4 time points, and stranger at the door observation (age 54 months only)	MacArthur health and behaviour questionnaire (HBQ).	Social competence at 12 years: ‘Never’ group (no DSED symptoms at any time): 58% were socially competent.	
				Youth risk behaviour survey.	‘Early’ group (DSED diagnosis before 54 months): 28% of children were socially competent. ‘Late’ group (DSED symptoms at 12 years only): 33% were socially competent. 'Persistent’ group: (DSED symptoms at *all* time points): 0% were socially competent.	
						
**2. Peer relationships**						
Bennet et al. (2009)	Qualitative	Social experiences of disinhibited children.	8 indiscriminate friendly children (aged 9–14 years, mean 11.5 years), with suspected/confirmed maltreatment history, recruited from clinical services and voluntary organisation for adoptive parents.	Semi‐structured interview with indiscriminate friendliness social scenarios (IPA analysis).	IPA themes: 1. difficulty with concepts of friendships. 2. Exclusion from peer friendships. 3. Need for trust in relationships. 4. parental attempts to instil stranger danger and 5. Kindness as a response from others (seeking kindness and acceptance).	32 High
			DSED symptoms were assessed using the standardised relationship problems questionnaire.			
Raaska et al. (2012)	Quantitative cross sectional	Bullying or victimisation in DSED^RAD^	364 international adoptees in Finland, ages 9–15 years (mean, 11.6 years) and comparison data of 146,767 children was derived from a large data set collected from Finnish schools.	Five to fifteen questionnaire	Children with mild DSED^RAD^ more likely to report victimisation. Children with severe DSED^RAD^ more likely to be both victims and bullies.	29 moderate
			Assessments conducted via postal survey (response‐rate 49.4%)	Olweus bully/Victim questionnaire (OBVQ).	Lack of social skills was associated with victimization but not independently from bullying.	
			Symptoms of DSED^RAD^ were measured via FINADO questionnaire, designed for use within the study.			
Kay and Green (2013)	Quantitative cross sectional	Assess DSED behaviours and associated functional impairment.in non‐institutionalised adolescents exposed to early maltreatment or neglect.	153 adolescents, at high risk of placement breakdown, referred by social workers, were assessed. The mean age was 174 months.	Health of the nation outcome scales for children and adolescents (HoNOSCA).	High risk group: Demonstrated significantly higher total and scale DAWBA‐RAD scores than the low risk group.	34 High
			Control group was a low risk community sample in a deprived area, recruited via schools and local youth clubs and mean age was 168 months.		The disinhibited indiscriminate scale (DAWBA‐RAD) was a significant predictor of impaired peer relationships on the HoNOSCA. The superficial relationships item showed the most association with functional impairment.	
			DSED symptoms measured via the standardised development and wellbeing assessment—reactive attachment disorder (DAWBA‐RAD).			
Guyon‐Harris, Humphreys, Fox, et al. (2019)	Quantitative cross sectional	Examine associations between signs of DSED and RAD and social functioning in early adolescence.	Post institutionalised children, randomised into a high quality foster care intervention (*n* = 55) compared to post‐institutionalised children in care as usual (*n* = 55) and 50 never institutionalised children from the local community. Participants assessed at 12 years.	Peer conflict scale (PCS).	Symptoms of DSED (and not RAD) were associated with greater caregiver perceptions of the child being victimised and were perceived to have greater conflicts in peer relationships.	35 High
			Signs of DSED or RAD were measured using the standardised disturbances of attachment interview‐ early adolescence.	MacArthur health and behaviour questionnaire (HBQ).	DSED (and RAD) associated with lower social competency, independent of placement disruption or time in institutional care.	
						
Seim et al. (2022)	Quantitative cross sectional	To assess possible co‐occurrence of psychopathology and/or psycho‐social problems in children with DSED, or RAD.	A total sample of 381 adolescents (mean age, 16.7, range 12–20 years) in a group residential setting were assessed and 31 presented with DSED.	Psycho‐social difficulties were assessed via the child behaviour checklist for ages 12–18 years,	Children with DSED had significantly greater number of associated psycho‐social problems than children without DSED, (mean 4.04) and were more likely to be bullied.	High 32
			DSED was assessed using the preschool age psychiatric assessment (PAPA) (caregiver report) due to lack of available age appropriate measure and then DSM‐5 criteria applied stringently to determine if symptoms met diagnostic criteria.	In addition, the child and adolescent psychiatric assessment was utilised to assess for ‘exposure to bullying’ (and other psychiatric problems, not relevant to this review).		
**3. Self‐esteem/Self‐concept**						
Vervoort et al. (2014)	Cross sectional quantitative	To compare indiscriminately friendly children with controls regarding their perceptions of self, reliability trust in significant others and perceptions of child‐teacher relationship.	33 likely cases for disinhibited reactive attachment disorder (d‐RAD, DSM‐IV) from special education for children with emotional and behavioural disorders (mean age, 8.52) and 33 controls from general education (mean age, 8.42) (matched by age, sex and socio‐economic status).	Self‐description Questionnaire‐I (SDQ‐I) and the following 2 scales were used:	Perceptions of self‐concept were higher in the d‐RAD group than those of the control. The d‐RAD group also reported more trust in the reliability of significant others but greater conflict with their teachers, despite dependency on the teacher‐child relationship.	28 moderate
			DSED symptoms were assessed using the standardised relationship problems questionnaire	The general‐self scale and the peer relations scale. Three domains are assessed: Cognitive competence, physical competence and peer acceptance.		
						
Vacaru et al. (2018)	Quantitative cross sectional	To investigate possible associations between disturbed attachment, (DSED & RAD) and self‐concept.	Thirty‐three institutionalised children (Mean age, 9.75, range 4–12 years) participated along with staff working at the institute (caregivers, social workers and teachers).	Self‐concept was assessed via the validated measure, the pictorial scale of perceived competence and social acceptance in young children.	DSED, and RAD, were generally associated with negative perceptions of self‐competence, but self‐perceptions of physical competence were higher than the teachers' perception of their physical competence.	High 33
			DSED was assessed via the disturbances of attachment interview (caregiver report) and the behavioural signs of disturbed attachment (observational measure)			
Seim et al. (2021)	Cross sectional quantitative	Investigate whether global and domain‐specific self‐esteem among adolescents living in group residential care differs between those with a RAD diagnosis, a DSED diagnosis, or neither RAD nor DSED diagnoses, and with adolescents in the general population.	306 individuals living in youth residential care in Norway, (mean age, 16.8 years, range, 12–20 years) of which 26 met diagnostic criteria for DSED and 28 RAD.	Self‐perception profile for adolescents (SPPA) measuring global self‐worth and domain‐specific elements of scholastic competence, social acceptance, athletic competence, physical appearance, romantic appeal, and close friends.	Children with DSED demonstrated lower self‐esteem regarding social acceptance compared to both children with RAD and the environmental control, as well as the ‘typical development’ group	37 High
			DSED/RAD symptoms were assessed via preschool age psychiatric assessment (PAPA) (caregiver report) due to lack of available age appropriate measure and then DSM‐5 criteria applied stringently to determine if symptoms met diagnostic criteria.			
**4. Social interaction**						
Groark et al. (2011)	Quantitative cross sectional	Assessed caregiver characteristics and child‐caregiver interactions in Latin american institutions.	3 institutions with 120 children, many of whom were indiscriminately friendly, were included. Age range, from birth to 7 years old (wards had average 8–23 children with some larger).	The caregiver—‐child social/Emotional/Relationship rating scale (CCSERRS): Designed for purposes of measuring child‐carer interactions in institutions.	CCSERRS: Low levels of carer responsiveness and availability to the child were rated, although interactions were more positive during free play and physical caring. Children demonstrated poor responsiveness/anticipation, child directed behaviours and relationship with the caregiver.	27 moderate
				Children's problem behaviour scale (CPBS).	CPBS: High levels of indiscriminate friendliness, non‐compliance, provocative interpersonal behaviour and aggression, but little stereotyped self‐stimulation or withdrawn behaviours.	
Sadiq et al. (2012)	Cross sectional quantitative	Pragmatic language deficits in children with DSED^RAD^ compared to children with ASD and typically developing children.	35 children with DSED^RAD^ (mean age, 6.7, range, 5–8 years); 52 children with autism (mean age, 6.4, range, 5–8 years) and 39 with typical development (TD) (mean age, 6.5, range 5–8 years) verbal IQ was within ‘normal’ range for all 3 groups.	Children's communication checklist (CCC).	CCC: The DSED^RAD^ and autism groups performed significantly more poorly on all sub‐scales except syntax and speech.	28 Moderate
			DSED^RAD^ group recruited from clinical and social work services, the TD group recruited via their general practitioners, during a previous study. The autism group were recruited from a specialist tier 4 clinic.		The DSED^RAD^ group performed most poorly in domains use of language in context, rapport and social relationships.	
					Only the DSED^RAD^ group differed from the TD group on rapport.	
						
Davidson et al. (2023)	Mixed method cross sectional	To determine the outcomes of children with autism, in comparison to children with DSED, on standardised autism measures and a socially challenging behavioural observational assessment called live.	10 children with autism (no maltreatment history), 8 children with confirmed symptoms of DSED who were either caregiver referred or referred by local mental health service, and 10 typically developing children who were caregiver referred (age range, 5–11 years and groups were matched by age).	Diagnostic interview for social and communication disorders (DISCO):	Disco: Almost all the children with DSED met core criteria for autism on the parent interview.	30 High
			DSED symptoms were confirmed via caregiver reports using standardised relationship problems questionnaire and reactive attachment disorder and disinhibited social engagement disorder assessment interview and via observation using the standardised waiting room observation.	Autism diagnostic observational Schedule‐2 (ADOS‐2)	ADOS‐2: 62.5% of children with DSED did *not* meet diagnostic criteria for autism. But false positive outcomes were found in 3 cases of DSED, who had additional moderate to severe symptoms of ADHD.	
				Live assessment: An unstructured dynamic assessment (2 assessors) designed to increase unpredictability and greater social challenge.	LIVE: The DSED group were more able than children with autism to engage in complex humour, their play was more creative and spontaneous, and children with DSED tended to involve the assessors, even if their interactions were not entirely appropriate.	
					Controlling and/or obsessive behaviours and lack of empathy were noted in both groups.	
Sheaffer, Golden, and Averett (2009)	Cross sectional quantitative	Decoding of emotions from facial expressions and paralanguage (intonation patterns) in DSED^RAD^	17 children with DSED^RAD^ 15 children in foster care, without DSED^RAD^ recruited by social services, private clinicians and university psychology department; 31 typically developing children recruited via an afterschool organisation and university psychology department (age range 5–19 years).	Diagnostic analysis of nonverbal accuracy (DANVA2): 2 sub‐tests relating to facial expressions, 1 sub‐test relating to child paralanguage and 1 to adult paralanguage.	No significant differences found between groups regarding ability to decode emotions from facial expressions or paralanguage.	26 Moderate

Abbreviations: CCAT, Crowe Critical Appraisal Tool; DSED, Disinhibited Social Engagement Disorder; IQ, intelligence quotient; RAD, Reactive Attachment Disorder.

Due to heterogeneity of study methods, we present the findings as a narrative synthesis. The four emergent sub‐themes were: 1. social competence (general); 2. peer relationships, 3. self‐esteem/self‐concept related to social functioning and 4. social interaction/communication skills.

### Social competence (general)

Two studies of moderate quality, investigated differences in total scores on standardised measures of problem and pro‐social behaviour. Children with DSED^RAD^ scored significantly more poorly regarding social behaviours, (conflicts and pro‐social) than typically developing children (Millward et al., 2006; Pritchett et al., 2013). In both of these studies, the authors did not discriminate between DSED and RAD and Pritchett et al., recognise that as UK norms were not available the study is limited by comparison to normative data of American children.

Another moderate quality study found that children with DSED who had mild intellectual difficulties performed significantly worse on the socialisation domain of the Vineland assessment compared to intellectually similar controls (Giltaij et al., 2016). The comparison group of children with similar intelligence quotient (IQ), but without DSED, is a relative strength, but the sample size of children with DSED (*n* = 7) was small and the authors recognised that some had mixed DSED and RAD.

In the final study, social competence was measured in 136 post‐institutionalised children at age 12 years. To meet threshold for social competency the child had to be competent in 6 of the following 7 domains: family relationships, peer relationships, physical health, mental health, academic performance, substance misuse and risky behaviour. Overall, children with symptoms of DSED were significantly less socially competent than those without (Guyon‐Harris, Humphreys, Fox, et al., 2019). When DSED symptoms were measured dimensionally (‘never’‐no symptoms, ‘early’‐ symptoms before 54 months, ‘late’‐ symptoms at 12 years and ‘persistent’‐ symptoms before 54 months and at 12 years), 57% of the ‘never’ group met competency threshold, compared to just 28% in the ‘early group, ‘33%’ in the late group and of most note, 0% in the ‘persistent’ group. This was a high quality study, which expanded on measurements of social functioning in DSED, and adds weight to the consistent reports of this theme; children with DSED appear to have poorer general social competence than typically developing peers.

### Peer relationships

A high‐quality qualitative study by Bennett et al. (2009) utilised rigorous Interpretative Phenomenological Analysis following a story task with a strong sample of eight indiscriminately friendly children, who had experienced childhood maltreatment. The inclusion of opinions of children with DSED was a strength, and demonstrated, lack of understanding about friendships, feelings of social exclusion and a perceived need for acceptance.

Kay and Green (2013) investigated DSED in a high‐quality case‐control study of a non‐institutionalised community sample of 153 high risk adolescents. Eighty‐nine met ‘caseness’ via a standardised measure, for what they termed Disinhibited Indiscriminate Symptoms. On the Health of Nations Outcome Scales, which was double‐rated blindly, the Disinhibited Indiscriminate Symptoms factor made an independent prediction of greater peer problems.

Raaska et al. (2012) included a large, sequentially sampled group of 364 adopted children with DSED^RAD^ compared to large‐scale register data. Twenty percent of children with DSED^RAD^ experienced victimisation, 8% bullied others and both bullying and victimisation were present independent of learning and language skills. Lack of social skills was also associated with victimisation. The large sample and consideration of possible confounding factors were strengths but due to lack of discrimination between DSED and RAD, it must be considered moderate quality.

Seim et al. (2022) also examined victimisation, bullying and aggression in a reasonable foster care home sample, (*n* = 31) and the findings support those of Raaska et al. This was a study of high quality which separately identified DSED and RAD. The age range (12–20 years) was slightly beyond the upper age limit of our search criteria (up to 18 years), but due to the scarcity of DSED research, it was thought more beneficial to include it, with acknowledgement of this contravention.

A second high quality study by Guyon‐Harris, Humphreys, Fox, et al. (2019) compared a sample of post institutionalised foster care children (*n* = 55) to a sample of post‐institutionalised children in care as usual (*n* = 55) and a group of children from the local community (*n* = 50). Although symptoms of both DSED and RAD were assessed, it was not clear the total number of children who met criteria for DSED. Bearing this in mind, symptoms of DSED, and not RAD, were associated with greater caregiver perceptions of victimisation (rejected/bullied), and children with DSED were perceived to have greater conflicts in peer relationships. There was no significant association between symptoms of DSED, victimisation and the teachers' perceptions.

### Self esteem/self concept in relation to social functioning

A case‐control study on self‐concept, (worthiness as a person and acceptance by peers), found that in 33 school‐aged children with Disinhibited RAD, their perceptions of self‐concept were higher than typically developing peers (*n* = 101) (Vervoort et al., 2014). This study was considered of moderate quality due to possible sampling bias and possible confounding variables such as co‐occurring neurodevelopmental conditions.

In contrast, a high‐quality study by Vacaru et al. (2018), found that DSED and RAD was associated with poorer self‐concept, (cognitive competence, physical competence and peer acceptance) in a reasonable sized sample of post‐institutionalised children (*n* = 33). However, self‐perception ratings of physical competence were greater than the teacher ratings. The main limitation was that results were not discussed as to how they relate to DSED and RAD individually.

A high‐quality study by Seim et al. (2021) also found that, in a community sample, children with DSED, (*n* = 26) demonstrated lower self‐esteem regarding social acceptance compared to children with RAD (*n* = 28), environmental controls, (*n*=) and typically developing children (stratified sample of 10,480 school children). The authors acknowledge that the lack of standardised measures of DSED for the age group was a limitation but used as close to age measures as possible and then stringently applied DSM‐5 diagnostic criteria. The upper age range of the sample (20 years) went just beyond our inclusion criteria (up to 18 years), but the mean age was within the limits (16.2. years). Again, it was felt more beneficial to include these findings while acknowledging the caveat.

Both high quality studies suggest that self‐esteem regarding social acceptance generally may be lower in children with DSED, but Vacaru and Vervoort's studies together perhaps suggest that in specific circumstances, children with DSED may perceive themselves as more competent than significant others see them.

### Social interaction skills

Groark et al. (2011) measured dyadic interaction between caregivers and indiscriminately friendly children (*n* = 123) in an institution using a measure designed for this environment. The institutionalised children showed little anticipation of care‐giver interactions and tended not to signal or direct interactions. These findings are considered in relation to caregiver behaviour which lacked empathy and showed low responsiveness to child initiations. Due to the unusual caregiving environment, findings have limited generalisability.

Sadiq et al. (2012) used the standardised parent report Child Communication Checklist to investigate the pragmatic language (use of social language in context) of a community‐based sample of children with DSED^RAD^ (*n* = 35), compared to children with Autism (*n* = 52) (average verbal IQ) and typically developing children (TD) (*n* = 39). The DSED^RAD^ and Autism groups significantly differed from the TD group in domains of, inappropriate initiation, coherence, stereotyped conversation and social interests, but *only* the DSED^RAD^ group significantly differed regarding rapport. Surprisingly, the DSED^RAD^ group showed *greater* impairment than children with Autism regarding use of language in context, rapport and social relationships. However, this is a study of moderate quality because the authors did not discriminate between DSED and RAD and unconfirmed co‐occurring Autism, based on parent report only, was a possible confounding variable.

In contrast, a high‐quality qualitative study reported that children with DSED (*n* = 8), compared to children with Autism (*n* = 10), demonstrated more engagement in complex humour, more creativity and spontaneous play and more often involved the assessors, even if their interactions were not entirely appropriate, during unstructured clinical observation (as opposed to caregiver report, as above.) Controlling and/or obsessive behaviours and lack of empathy were observed in both groups (Davidson et al., 2023). ADHD was co‐existing in some of the children with DSED and impacted social behaviour on the standardised ADOS‐2 assessment, but social skills were less effected by ADHD during the unstructured observation. The main limitation was the smaller sample size.

Finally, Sheaffer, Golden, and Averett (2009) investigated ability to decode emotions from facial expressions and paralanguage in children with DSED^RAD^ (*n* = 17) compared to a foster care group (*n* = 15), without DSED^RAD^, and a typically developing group (*n* = 31) and found no group differences on the standardised measures. This study was considered of moderate quality because they did not discriminate between DSED and RAD, the samples were small and the DSED^RAD^ group were receiving therapy, which could inadvertently influence results.

The findings within this theme are mixed. Each study is measuring slightly different aspects of social interaction/communication which may account for some differences, and it appears that type of measurement (caregiver vs. observation) may be important.

## DISCUSSION

This systematic review aimed to address the gap in knowledge regarding the social functioning of children with DSED. It is recognised that children who have experienced early childhood maltreatment are at higher risk of social relationship and communication difficulties (Cicchetti, 2016), and it seems that children with DSED are no exception. Regarding general social competencies, reports were consistent; children with DSED may present with greater social functioning difficulties than peers (Giltaij et al., 2016; Guyon‐Harris, Humphreys, Fox, et al., 2019; Guyon‐Harris et al., 2019, 2019; Millward et al., 2006; Pritchett et al., 2013), which supports the reconceptualization of DSED as a disorder of social‐relatedness, separate from RAD (DSM‐5). It is perhaps unsurprising then that children with DSED appear to be at higher risk of peer victimisation and conflicts in peer relationships (Guyon‐Harris, Humphreys, Fox, et al., 2019; Kay & Green, 2013; Raaska et al., 2012; Seim et al., 2022). Only one study included child report, as opposed to caregiver report, but this qualitative study demonstrated that lack of understanding of friendships (Bennett et al., 2009), may be a key area for further investigation. For example, are peer problems reflective of cognitive deficits, as associated with Autism, or is lack of understanding of these relationships arising from missed opportunities and stressful experiences, as proposed by McCrory et al. (2022)? The findings regarding the social interactions/communication of children with DSED were mixed, but differences in measurement stood out as an important factor. However, one interesting point of convergence between the findings of Sadiq et al. (2012) and Davidson et al. (2023) regarded the initiations of children with DSED, which were not always appropriate, even if Davidson et al. did not perceive them as autistic in nature. Moreover, Davidson et al. found that controlling behaviours and lack of empathy overlapped between DSED and Autism. These latter behaviours, in addition to inappropriate initiation, are likely to impact rapport, one of the key areas of difficulty reported by Sadiq et al. It is conceivable that subtle pragmatic language/interaction skills are negatively impacted by both core symptoms of DSED and/or these additional behaviours, which have been reported in other studies (Mukaddes, et al., 2000; Pears et al., 2010; Rutter et al., 1999). Future research is required, with larger samples, and would benefit from both caregiver report *and* observation, perhaps involving relevant professionals like Speech and Language Therapists to complete targeted investigation of pragmatic language. It is also vital that observational studies, such as Davidson et al.’s, be repeated, but in comparison to typically developing children. The interactional skills of the children with DSED may be ‘improved’ compared to the children with Autism, but it is unclear if the skills of the DSED group were developmentally appropriate.

Finally, most of the included studies suggest that children with DSED may have poorer self‐estem/self concept with regards to social acceptance (Seim et al., 2021; Vacaru et al., 2018), but one study found that children with DSED had higher perceptions of self‐concept than typically developing peers (Vervoort et al., 2014). As participants in the Seim et al. study were older (mean age, 16.5 years), it is plausible that by late adolescence, when peer relationships have even greater salience in identity formation and social behaviour (Merritt & Snyder, 2015; Reitz et al., 2014) participants were more acutely aware of their difficulties. Yet, Vacaru et al. (2018) did find that in one specific domain, physical competence, children with DSED perceived themselves as more competent than significant others perceived them. The authors argue that a self‐perception bias may have some benefit for socialisation in certain settings (Vacaru, et al., 2018), and this seems worthy of further exploration. For example, other groups of children with neurodevelopmental conditions, such as ADHD, have been found to present with self‐perception bias regarding competencies, which have been considered self‐protective (Ohan and Johnston (2002)). As 40% of Vervoort's sample were found to have co‐occurring neurodevelopmental conditions, it would be useful to better understand whether sample bias is accounting for Vervoort's findings or whether, in some areas of socialisation, self‐perception bias may also be protective for children with DSED.

### Neurodevelopmental complexity and future research

Both Autism and ADHD are associated with poorer social functioning and poorer peer relationships, however, symptoms of both were found to overlap in some of the included studies (Davidson et al., 2023; Sadiq et al., 2012; Vervoort et al., 2014). Although there appears to be no aetiological reason why DSED and Autism cannot co‐exist (Mayes et al., 2017; Minnis et al., 2020), there are now some studies suggesting that core symptoms of DSED are discriminable from Autism (Davidson et al., 2015, 2023; Davidson, Minnis and Moran, 2022; Rutter et al., 1999). In contrast, core symptoms of ADHD, such as poor inhibitory control appear to be associated with core symptoms of DSED (Bruce et al., 2009; Pears et al., 2010). Preliminary research suggests that neglect is associated with negative impact on higher cognitive skills (McLaughlin, 2017), therefore further research with children with DSED may help to elucidate understanding regarding possible pathways into DSED and why DSED is more persistent in some children with DSED than others (Scheper et al., 2019). In this review, Guyon‐Harris, Humphreys, Fox, et al. (2019) found that children with *persistent* DSED had the greatest social difficulties (0% were socially competent), but it is unknown whether there were any differences within the ‘persistent’ DSED group, regarding neurodevelopmental complexity. Further understanding of the inter‐play with ADHD symptoms is important for case management as adolescents with DSED in residential care were 2.5 times more likely to have additional ADHD (Seim et al., 2022) and longitudinal data from the English‐Romanian Adoptees studyde monstrated that at 15 and 25 years old, DSED behaviours were still present, with some overlap with Autism and/or ADHD (Kennedy et al., 2017; Sonuga‐Barke et al., 2017), but at 25 years, functional problems, like employment issues, were related to the inter‐play with ADHD (Kennedy et al., 2017).

Gajwani and Minnis (2023) argued that children with DSED, or RAD, may experience ‘double jeopardy’ regarding mental health outcomes due to interplay of co‐occurring neurodevelopmental conditions. Our findings appear to suggest that children with DSED are at higher risk of social problems, therefore in cases of childhood maltreatment, *both* DSED and possible overlapping neurodevelopmental conditions must be considered alongside impaired social function to provide a fuller picture for health and social care management. Furthermore, social problems need to be considered in the *early years*, and as a preventative approach to later mental health difficulties, especially given the persistent nature of DSED.

### Limitations

Half of the studies were considered of moderate quality due to small samples, possible confounding variables and, in some cases, lack of discrimination between DSED and RAD. Thus we have been careful in our discussion to present only hypotheses and suggest some caution in interpretation of findings. It was also noted that one relevant study (Vacaru et al., 2018), was initially missed out. This occurred because the abstract referred to disturbed attachment and exploratory behaviours, thus we wrongly assumed that the study was about attachment *patterns* rather than DSED. On noting this error, the study was read in full and subsequently included. Due to the scarcity of social relationship literature regarding DSED, we took a top‐down approach to the search, focussing on broad relational terms and considered possible themes as findings emerged. This meant the search did not include the key words, ‘self esteem’/‘self concept’ and inclusion of these terms within future investigations may yield an even more inclusive picture.

### Conclusion

Bearing in mind the limitations, the evidence consistently suggests that children with DSED present with poorer social competencies than peers, have greater peer difficulties and may have poor self esteem/self concept. Further research in specific areas such as pragmatic language and regarding the interplay with other co‐occurring neurodevelopmental conditions is required. However, researchers and clinicians need to consider the presence of DSED, in maltreated children, possible neurodevelopmental overlap *and* relative impact on social functioning to better support this underrepresented group of children and their families.

## AUTHOR CONTRIBUTIONS

**Claire Davidson**: Conceptualisation, methodology, investigation, analysis, writing, writing‐review and funding acquisition. **Shahela Islam:** Investigation, analysis and writing‐review. **Enrico Venturini**: Investigation and writing‐review. **Anja Lowit:** Conceptualisation, methodology, supervision, review of writing. **Christopher Gillberg**: Conceptualisation, methodology, supervision, review of writing. **Helen Minnis**: Conceptualisation, methodology, supervision, review of writing.

## CONFLICT OF INTEREST STATEMENT

The authors have declared they have no competing or potential conflicts of interest.

## ETHICS STATEMENT

Ethical approval was not required for this article.

## Data Availability

Data derived from public domain resources.
